# The Influence of the Addition of Basalt Powder on the Properties of Foamed Geopolymers

**DOI:** 10.3390/ma17102336

**Published:** 2024-05-14

**Authors:** Michał Łach, Barbara Kozub, Sebastian Bednarz, Agnieszka Bąk, Mykola Melnychuk, Adam Masłoń

**Affiliations:** 1 Faculty of Material Engineering and Physics, Chair of Material Engineering and Physics, Cracow University of Technology, Jana Pawła II 37, 31-864 Cracow, Poland; michal.lach@pk.edu.pl (M.Ł.);; 2 Department of Materials Science, Lutsk National Technical University, Lvivska 75, 43000 Lutsk, Ukraine; 3 Department of Environmental Engineering and Chemistry, Rzeszow University of Technology, Powstańców Warszawy 6, 35-959 Rzeszów, Poland

**Keywords:** alkali-activated materials, foamed geopolymers, basalt powder, waste materials, insulating materials

## Abstract

Geopolymers are binder materials that are produced by a chemical reaction between silica or aluminum compounds with an alkaline activating solution. Foamed geopolymer materials are increasingly being cited as a viable alternative to popular organic insulation materials. Since the foaming process of geopolymers is difficult to control, and any achievements in improving the performance of such materials are extremely beneficial, this paper presents the effect of the addition of basalt powder on the properties of foamed geopolymers. This paper presents the results of physicochemical studies of fly ash and basalt, as well as mechanical properties, thermal properties, and structure analysis of the finished foams. The scope of the tests included density tests, compressive strength tests, tests of the thermal conductivity coefficient using a plating apparatus, as well as microstructure tests through observations using light and scanning microscopy. Ground basalt was introduced in amounts ranging from 0 to 20% by mass. It was observed that the addition of basalt powder contributes to a reduction in and spheroidization of pores, which directly affect the density and pore morphology of the materials tested. The highest density of 357.3 kg/m^3^ was characterized by samples with a 5 wt.% basalt powder addition. Their density was 14% higher than the reference sample without basalt powder addition. Samples with 20 wt.% basalt addition had the lowest density, and the density averaged 307.4 kg/m^3^. Additionally, for the sample containing 5 wt.% basalt powder, the compressive strength exceeded 1.4 MPa, and the thermal conductivity coefficient was 0.1108 W/m × K. The effect of basalt powder in geopolymer foams can vary depending on many factors, such as its chemical composition, grain size, content, and physical properties. The addition of basalt above 10% causes a decrease in the significant properties of the geopolymer.

## 1. Introduction

Geopolymers are materials of increasing interest to the scientific community and the construction industry. They have been studied for several decades [1,2,3]; however, many aspects related to improving their properties remain undiscovered. Geopolymer materials are a very interesting and attractive material; however, comparing their potential 20 years ago, it must be stated that the situation regarding the feasibility of their implementation has changed significantly. More and more often, scientists present critical comments regarding geopolymerization technology. This is related to certain limitations such as difficulties in eliminating efflorescence and the fact that this technology is very sensitive to changes in raw material prices, and in recent years, up to 300% increases in the price of sodium hydroxide have been observed. Most research, publications, and first implementations have been carried out for geopolymers produced based on fly ash and slag. It should be borne in mind that these raw materials are no longer as available as they were in the 1980s, 1990s, and at the beginning of the 21st century. Many steel mills are closed in Europe and production has been moved to Asia. The situation is similar for coal combustion and fly ash. The current policy of many countries is to abandon coal burning, which results in smaller amounts of available fly ash.

Therefore, advanced research and the search for alternative sources of waste raw materials are of great importance for the further development of geopolymers.

Geopolymer foams are a special type of porous material that is produced by a chemical reaction between silica or aluminum materials and an alkaline activating solution [1]. This process leads to the formation of a three-dimensional polymer network, which provides the structure of the material [4]. Geopolymer foams are used in a variety of construction applications, including as insulation materials, structural materials, and civil engineering [5]. Geopolymer foams are typically lightweight, which makes them attractive for lightweight applications such as thermal insulation and lightweight structural components. Despite their low density, geopolymer foams have good mechanical strength. Thanks to their porous structure, geopolymer foams have good insulating capacity. They are often resistant to many chemicals [6,7,8,9,10,11]. Due to their chemical composition and structure, geopolymer foams can exhibit good heat resistance. Many geopolymer foams are made from natural raw materials, making them more environmentally friendly than some traditional building materials [6].

Despite the continuous development of these materials and the numerous scientific studies conducted in the area of the effect of ceramic additives on their properties, there is still much to be discovered to make their implementation more feasible. Continuous efforts should be made to develop compositions with the least possible environmental impact and ease of large-scale application. It is known that the most commonly described raw materials thus far, such as fly ash and blast furnace slag in many countries, are already scarcely available, and other alternative waste resources should be sought. All over the world, due to environmental policies, coal combustion is being abandoned, and heavy industry generates slags, which are largely used in cement plants. However, it should be remembered that the mining industry still generates quite a lot of waste raw materials from the extraction and processing of rocks. One such example is basalt powder [12,13,14].

Basalt powder is a natural product extracted from basalt volcanic rocks. It has many properties that make it a popular additive in many fields, such as agriculture, horticulture, construction, and industry. The chemical composition of basalt powder is as follows: (SiO_2_) 46.6%, aluminum oxide (Al_2_O_3_) 14.3%, iron oxide (Fe_2_O_3_) 11.4%, calcium oxide (CaO) 9.21%, magnesium oxide (MgO) 7.90%, sodium oxide (Na_2_O) 3.10%, and titanium oxide (TiO_2_) 1.95%. In addition, potassium oxide (K_2_O) 0.823%, phosphorus oxide (P_2_O_5_) 0.48%, and manganese oxide (Mn_2_O_3_) 0.266% are present in amounts of less than one percent [15,16].

The powder in its composition also contains micronutrients, including iron, magnesium, manganese, zinc, boron, and silicon. Due to its calcium carbonate content, it is widely used in horticulture. Nevertheless, the main reason for its use is to balance the pH of soils. Another feature of this natural product is water absorption. This factor can be considered negatively or positively depending on the use of basalt powder. In agriculture and horticulture in the broadest sense, this ability is considered as a favorable aspect for growing plants and vegetables, since it facilitates the plants’ access to water. When used as a material for concrete or geopolymers, this property can be problematic due to the risk of having to use larger amounts of liquid activating solutions [17,18,19].

Due to its chemical properties, it is increasingly noted that basalt powder can be used to produce geopolymers or also to form an alkaline solution, the so-called alkaline activator, due to the presence of calcium carbonate, which acts as a catalyst during the geopolymer formation process. However, focusing attention on its use in geopolymer formation and its effect on strength, as well as mechanical properties, it is important to note its porous structure. The porosity of the structure affects absorptivity and permeability, as well as mechanical strength. The use of basalt powder is also ecologically advantageous because, as a natural product, it is not harmful to the environment [20,21].

The potential applications of foamed geopolymers based on fly ash and basalt powder are wide and cover many areas of construction. Therefore, they are one of the more interesting materials to look at in the context of modern and sustainable construction [22,23].

**Figure 1:**
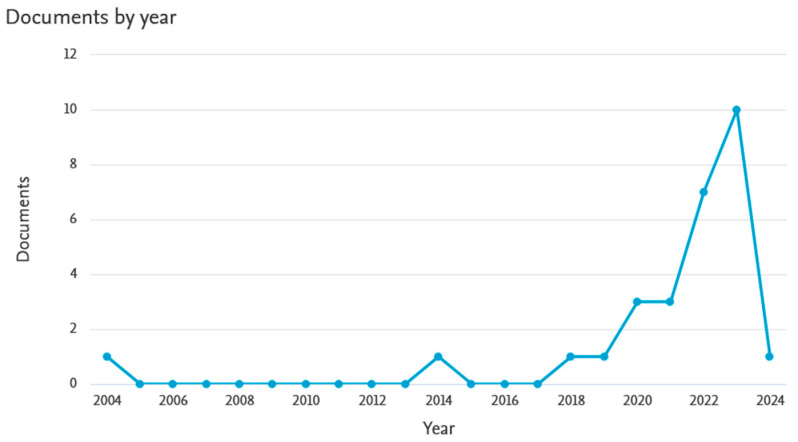
Analysis of the number of publications registered in the Scopus database on the introduction of basalt materials in powder form (crushed basalt) into geopolymer composites.

**Figure 2:**
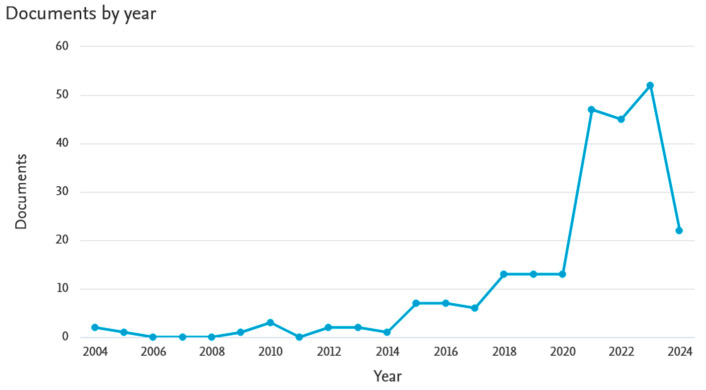
Analysis of the number of publications registered in the Scopus database on the introduction of basalt materials (mainly fibers) into geopolymer composites.

Despite the interesting properties of basalt and the existence of a large amount of waste basalt material, no attempt has been made thus far to use it as a powdered material to improve the performance of geopolymers. Basalt fibers are of greatest interest as a reinforcing material for various types of composites. The Scopus database registers as many as 237 articles from 2004 to 2024 on the introduction of basalt fibers into the geopolymer matrix, while only 28 articles deal with the introduction of basalt in powdered form. Figure 1 and Figure 2 below show an analysis of publications on this topic (according to the Scopus database). The number of articles regarding 2024 is incomplete because only 2 months of this year were covered by the analysis. Examples of the use of basalt fibers in geopolymer composites as reinforcement are widely reported in the literature [24,25,26,27]. However, due to its chemical composition, basalt in powdered form can also be used as an additive to improve the performance of foamed geopolymers.

As analysis in the SCOPUS database showed, the addition of powdered basalt or basalt fibers to foamed geopolymers was described in only four articles (search criteria words: basalt AND powder AND geopolymer AND foam) [28,29,30,31], of which only one was about shredded material introduced as an additive to foamed geopolymers. A careful analysis using various keywords found a few more scientific papers, such as [32,33]. The addition of basalt powder to geopolymers or cement mortars was also presented in articles by other authors [34,35,36,37]. The authors of these works confirmed the validity of using basalt powder for the production of cement mortars or geopolymers [38,39]. It has also been confirmed experimentally that basalt powder has a positive effect on the consistency of fresh cement composites and the strength of the cured composite [40]. As for geopolymers, it has been possible to successfully synthesize geopolymers from basalt by activation with sodium hydroxide solutions [35]. In that study, XRD analysis of various minerals found in basalt showed that basalt moderately reacted and dissolved with sodium hydroxide. Consequently, particles of the residue were placed as fillers in geopolymer matrices. The geopolymerization reactions occurred on the basalt surface, and the unreacted basalt particles play a supporting role in the geopolymer properties.

Since the number of scientific articles related to the introduction of powdered basalt into foamed geopolymers is small, and there is an identified knowledge gap in this area, it was decided to conduct a study involving the introduction of basalt powder additives into geopolymer paste in amounts ranging from 0 to 20% by mass, and then subject such compositions to foaming. This article presents the results of this research. The purpose of the study was to demonstrate the effect that different additions of basalt powder have on the properties of foamed fly ash-based geopolymers. The innovation of the research is mainly the introduction of a basalt additive into the composition of foamed geopolymers and the determination of the effect of this additive on density, thermal conductivity, compressive and tensile strength, and microstructure. As the analysis of the literature shows, the described research is innovative and very relevant to the possibility of implementing such materials. Analysis of the literature showed that the topic of basalt addition to geopolymer composites became particularly attractive from 2019 to 2020, and since then there has been a significant increase in the number of scientific reports in this area.

The novelty and innovation of the research presented in this article lie in the introduction of powdered basalt into foamed geopolymers simultaneously with stabilizers of the structure of foamed geopolymers. Since there is currently no knowledge on the impact of basalt addition on foamed geopolymers, this article should be treated as an impulse for further, more detailed research. As the results below show, the addition of basalt has a positive effect on the properties of geopolymers only up to a certain amount of addition, beyond which the properties of the geopolymer mixture decrease. Therefore, further research is necessary and the presented research results are new knowledge contributing to the development of geopolymer materials, and thus far this aspect has not been solved and presented in the scientific literature.

## 2. Materials and Methods

Fly ash from the Skawina Combined Heat and Power Plant (Skawina, Poland) was used to produce geopolymer foams. The basalt powder used in the study came from NB Minerals (Tychy, Poland). Oxide chemical composition analysis was performed for the base materials, namely, fly ash and basalt powder. XRF oxide analysis was performed on a SCHIMADZU EDX-7200 (SHIMADZU Europa GmbH, Duisburg, Germany). The test was carried out in an air atmosphere with holders designed for bulk materials and Mylar film, and the results are shown in Table 1.

**Figure 3:**
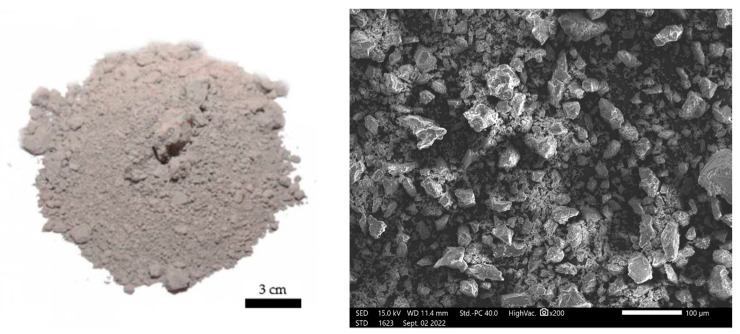
SEM microstructure of the fly ash used in the study and visualization of the form in which it was used.

**Figure 4:**
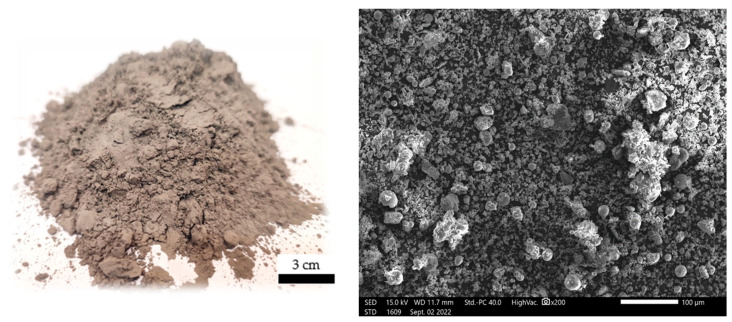
SEM microstructure of the basalt (basalt powder) used in the study and visualization of the form in which it was used.

Both fly ash and basalt powder were applied to the mixture in powder form (these materials were not mechanically processed, but were applied as supplied). Figure 3 and Figure 4 show a sample of fly ash and basalt powder. Photographs of particle morphology and others shown later related to the morphology of the finished composites were taken using a JEOL IT200 scanning electron microscope (JEOL, Akishima, Tokyo, Japan). Macroscopic images, on the other hand, were taken on a Keyence VHX-7000 digital optical microscope (KEYENCE INTERNATIONAL, Mechelen, Belgium).

**Figure 5:**
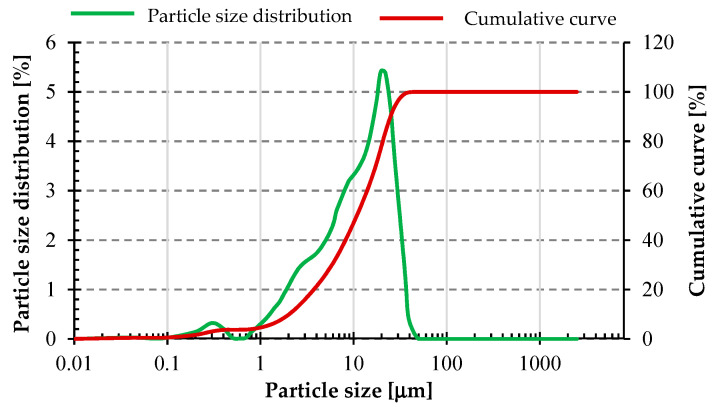
Particle size distribution of fly ash used in the study.

**Figure 6:**
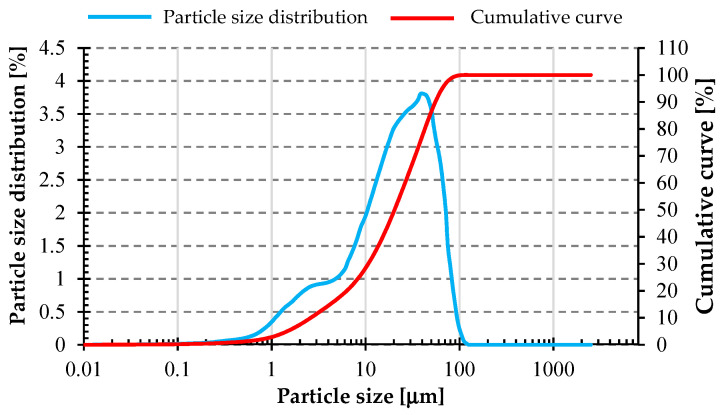
Particle size distribution of basalt powder used in the study.

Figure 5 and Figure 6 below show the particle size distribution of fly ash and basalt powder, respectively. These materials were not subjected to additional mechanical treatment before. A similar grain size distribution can be seen for both materials. The fly ash had a slightly higher degree of fineness. Measurement of the particle size distribution was carried out using a laser particle size analyzer from Anton Paar GmbH model PSA 1190 (Anton Paar GmbH, Graz, Austria) using Kalliope Professional software (version 2.22.1).

Table 2 below shows the grain size parameters of the raw materials used in the study. The values of D_10_ for both materials were on a similar level. However, the values of D_50_ and D_90_ were already about twice as small for fly ash compared to basalt powder.

To determine the phase composition of the fly ash and basalt powder samples used for analysis, the X-ray analysis technique (XRD) was applied. An X-ray diffractometer (PANalytical Aeris, producer; Malvern PANalytical (Lelyweg 1, Almelo, The Netherlands)) was used to carry out the study. Rietveld phase analysis was performed using the PDF-4+ database provided by the International Center for Diffraction Data (ICDD).

**Figure 7:**
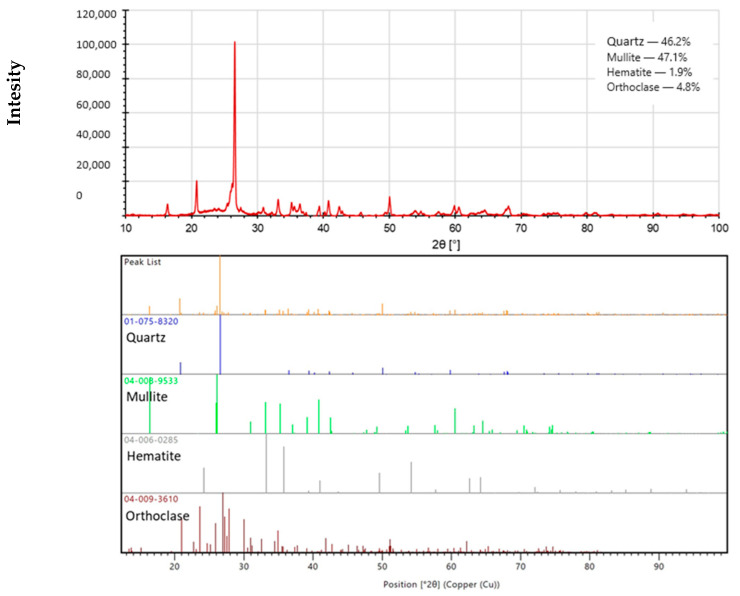
Diffractogram of the fly ash used in the study.

**Figure 8:**
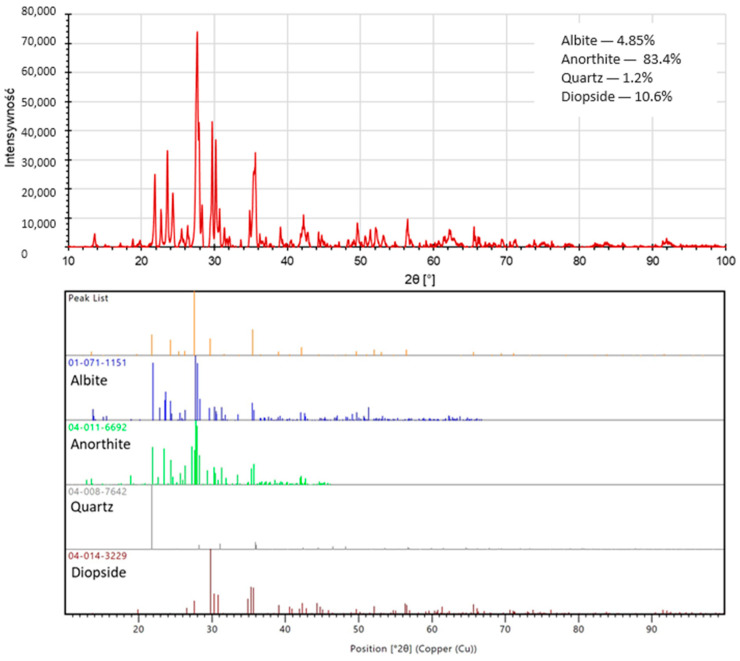
Diffractogram of the basalt powder used in the study.

Figure 7 and Figure 8 below show the phase composition of the raw materials used in the study. Based on the XRD phase analysis of the fly ash, it was determined that the fly ash used to produce the geopolymer composites studied in this paper consisted mainly of mullite (47.1 wt.%) and quartz (46.2 wt.%), with minor contributions from hematite (1.9 wt.%) and orthoclase (4.8 wt.%). In the case of basalt powder, XRD analysis conducted showed that the main phases of which it was composed were mainly anorthite (83.4 wt.%), as well as diopsite (10.6 wt.%), albite (4.8 wt.%), and quartz (1.2 wt.%). Both oxide composition and phase composition studies confirmed that fly ash and basalt powder have a suitable chemical composition and can be used as geopolymerization precursors, because they contain the appropriate amount of both SiO_2_ and Al_2_O_3_ and the molar ratios of these compounds are consistent with the literature [1,3,35,36]. Their degree of fineness is also suitable for carrying out alkaline activation processes.

For the recorded diffractograms, calculations were made of the amount of the amorphous phase in the form of amorphous silica. Calculations were performed in the MAUD program (Version 2.998; Made on a Mac). For the fly ash sample, the amount of amorphous silica was 29.81%, while the basalt sample contained 67.32% of amorphous silica.

Five variants of samples of geopolymer compositions with additives of fly ash and basalt powder were prepared for further studies, the proportions of which, along with the designations, are shown in Table 3. Basalt powder was added, increasing its amount by 5% by mass to approximately determine its optimal proportion. The basalt powder was dosed by precise mass measurement and introduced into the fly ash for uniform mixing.

Foam stabilizers in the form of putty gypsum (Dolina Nidy, Pińczów, Poland) and cellulose–hydroxyethyl cellulose (Glentham Life Sciences, Corsham UK) were used to prepare compositions with fly ash and basalt powder. Stabilizing additives ensure that the porous structure remains for a longer time immediately after foaming and the hardening process is faster. A 10 M solution of sodium hydroxide (NaOH) and an aqueous solution of sodium silicate R-145 (water glass) (molar modulus 2.5; density about 1.45 g/cm^3^), in a volume ratio of 1:2.5, were used as the activating solution. Hydrogen peroxide 36% was used as the foaming agent. The amounts of added ingredients are shown in Table 4 below.

The manufacture of geopolymers consisted of mixing the solid components and introducing the appropriate amount of additives, followed by dosing the alkali solution. After a dense plastic consistency was obtained (after about 15 min of mixing in a high-speed mixer), the foaming agent, which was hydrogen peroxide with a concentration of 36%, was dosed. Immediately after the addition of the foaming agent, the geopolymer mass was transferred to molds, sealed against moisture loss, and placed in a laboratory dryer. The curing process of the geopolymer composites was carried out at 75 °C for 24 h in an SLW 750 STD laboratory dryer (POL-EKO-APARATURA, Wodzisław Śląski, Pol-ska). After 24 h of annealing, the samples were taken out and unmolded. In each case, enough samples were produced to allow for at least five replicates. The samples were then properly prepared for further testing.

**Figure 9:**
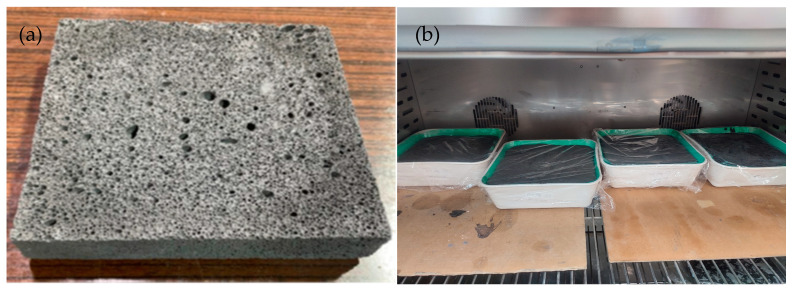
Example of manufactured geopolymer with basalt powder—sample for testing of thermal conductivity coefficient (80FA_20BF) (**a**); sample manufacturing method—formed samples in a chamber (**b**) (samples with dimensions of 20 × 20 × 3 cm).

Thermal conductivity tests were carried out using the HFM 446 Lambda Series from NETZSCH (Netzsch GmbH & Co., Selb, Germany). The measurements were carried out in a temperature range of 0–20 °C. Panels with dimensions of 20 × 20 × 3 or 4 cm were prepared for the tests. An example of the appearance of the test specimen and how it was formed are shown in Figure 9 below.

Compressive strength tests were carried out on a Matest 3000 kN concrete compression press (Matest, Treviolo, Italy) equipped with compression strength measuring heads and an additional force sensor up to 300 kN. The measurement was made at a speed of 0.05 MPa/s. The tests were carried out in accordance with PN-EN 12390-3:2019-07 [41]. Bending strength tests were carried out on rectangular specimens measuring 4 cm × 4 cm × 18 cm using a three-point bending test device, an Autograph AGS-X universal testing machine (Shimadzu, Kyoto, Japan), with a measuring range of up to 10 kN. The test speed was 5 mm/min, and the support spacing was 150 mm. The tests were conducted in accordance with PN-EN 12390-5:2019-08 [42].

## 3. Results

**Figure 10:**
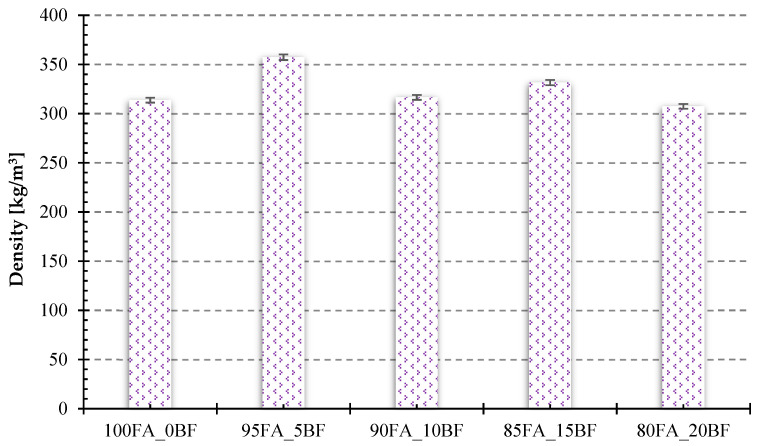
Results of a density study of foamed geopolymers made from fly ash with different proportions of basalt powder.

The density was read from a heat conduction coefficient tester. The density was determined automatically using a geometric method by measuring mass and volume. Figure 10 below presents the results of testing the density of the produced lightweight fly ash-based geopolymer structures with different proportions of basalt powder. The highest density of 357.3 kg/m^3^ was characterized by samples with a 5 wt.% basalt powder addition. Their density was 14% higher than the reference sample without basalt powder addition. Samples with 20 wt.% basalt addition had the lowest density, and the density averaged 307.4 kg/m^3^. No linear dependence of the density of the produced samples on the amount of introduced basalt was observed. This is most likely because the addition of basalt powder did not directly affect the density of the composition due to the similar density of the raw material itself to fly ash, but the addition did affect the consistency of the geopolymer mass and its ability to form a porous structure, as well as its stability. 

**Figure 11:**
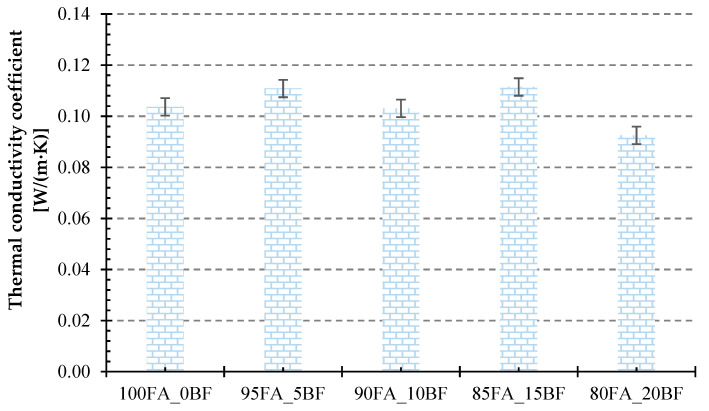
Thermal conductivity values for foamed geopolymers with different basalt powder content.

Figure 11 below shows the results of heat transfer coefficient measurements for the geopolymer foams produced. Very similar results were observed, as the obtained values did not differ significantly from each other regardless of the amount of basalt introduced. The highest values of the thermal conductivity coefficient were characterized by samples with 5 wt.% and 15 wt.% basalt additions, and these values were 0.11082 W/m × K and 0.11142 W/m × K, respectively. Comparing these values with the density test results shown in Figure 10, the dependence of the thermal conductivity coefficient on the density of the material is apparent, a dependence that is quite obvious and has been proven in many previous studies by other authors [7,8,9,10,11]. The sample with the lowest value of the thermal conductivity coefficient had 20 wt.% basalt mica addition and this value was 0.09254 W/m × K. The obtained results of 0.100 W/m × K are not competitive with commonly used insulation materials for construction, such as polystyrene and polyurethane foams or various types of wool, but it should be kept in mind that geopolymers are non-flammable materials capable of carrying loads even up to temperatures exceeding 1000 °C. They also have many other advantages. The literature reports that by properly controlling the foaming process of geopolymers, it is possible to obtain about twice as good results in terms of insulating properties. However, the purpose of the conducted research was not to obtain the best possible insulating parameters but to determine the suitability of the addition of basalt powder and its effect on the properties of foamed geopolymers. Obtaining very lightweight foamed structures with good insulating parameters involves a certain compromise related to their mechanical properties. Foamed geopolymers do not have to find application only in thermal insulation in construction, but can be used industrially to insulate various types of high-temperature installations. The experience of the authors of this article shows that, compared to other insulation materials commonly used, geopolymers do not lose their insulating properties as the temperature increases.

**Figure 12:**
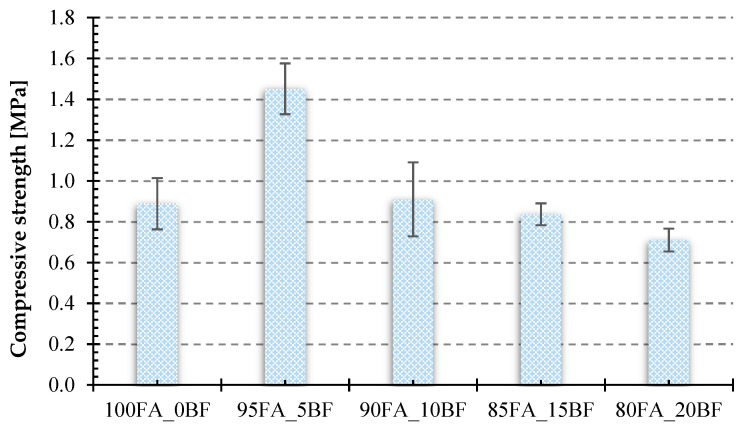
Compressive strength of geopolymer samples with different contents of basalt powder.

Figure 12 below shows the results of the compressive strength tests of manufactured geopolymer foams with basalt powder added.

As can be seen from the graph shown in Figure 12, which compares the compressive strength results for geopolymers with different contents of basalt mica, the material with 5 wt.% basalt addition—sample 95FA_5B—had the highest strength. The strength of this material was higher than that of the reference material by about 63%. In addition, only for this sample, the compressive strength exceeded 1 MPa, which means that it met the requirements of the JC/T2200-2013 [43] standard defining acceptable compressive strengths of foam insulation panels made of ordinary Portland cement [5]. The sample with a 20 wt.% addition of basalt powder had the lowest strength. Its compressive strength was lower than that of the reference sample by about 8%. A linear decrease in compressive strength with an increase in the proportion of basalt powder is evident.

**Figure 13:**
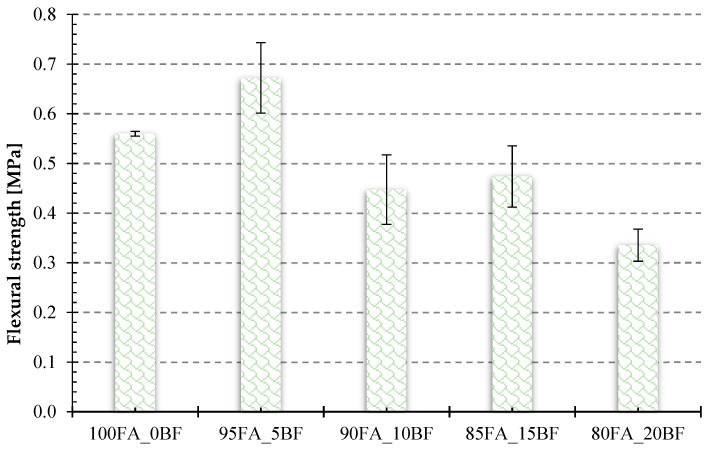
Flexural strength of geopolymer specimens with different contents of basalt powder.

Comparing the flexural strength results shown in Figure 13, significant discrepancies in the flexural strength values for the test specimens were also apparent here. The highest flexural strength value was characterized by the sample with 5 wt.% basalt addition. Its flexural strength was 0.67 MPa. Table 5 shows all the average numerical values for the results obtained in the flexural and compressive strength tests.

A comparison of the test results presented in Figure 12 and Figure 13 confirms that the best strength properties were obtained with the addition of 5% basalt. The addition of 5% basalt powder increased both the bending strength and compressive strength. Increasing the addition of basalt powder resulted in a decrease in compressive and bending strength, which was particularly visible in samples with 15% and 20% of basalt powder added.

**Figure 14:**
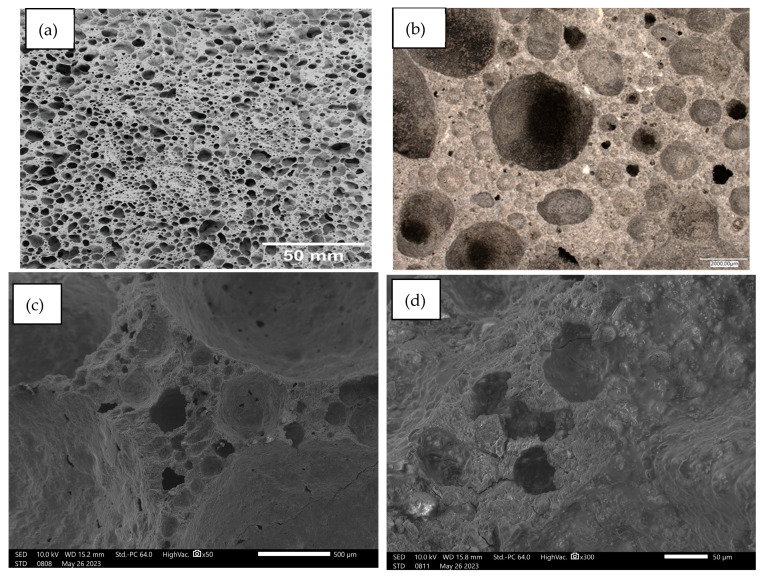
Images of the microstructure of foamed geopolymer 100FA_0BF: (**a**,**b**) macrophotographs taken with an optical microscope; (**c**,**d**) microphotographs taken with a scanning electron microscope.

**Figure 15:**
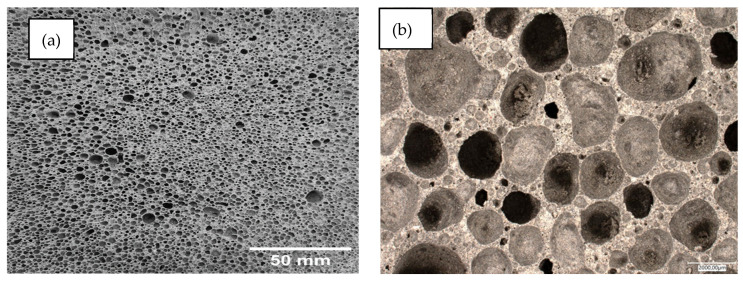

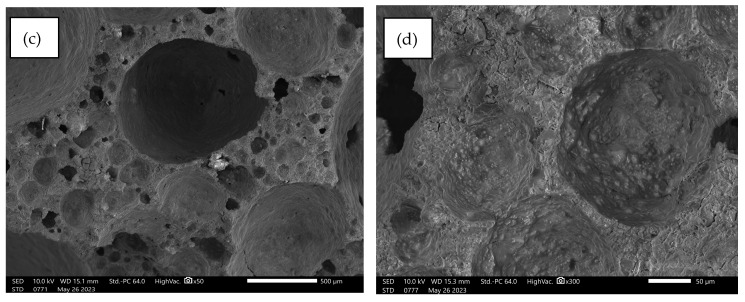
Images of the microstructure of foamed geopolymer 95FA_5BF: (**a**,**b**) macrophotographs taken with an optical microscope; (**c**,**d**) microphotographs taken with a scanning electron microscope.

**Figure 16:**
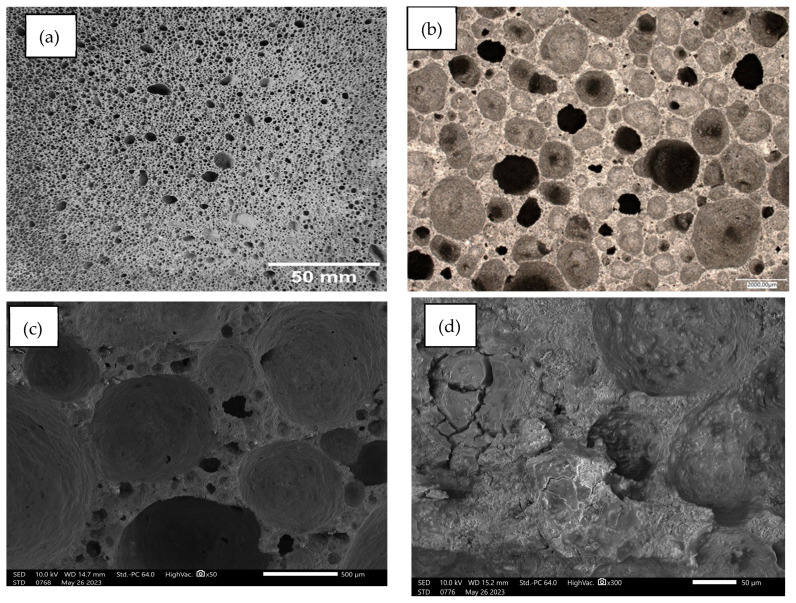
Images of the microstructure of foamed geopolymer 90FA_10BF: (**a**,**b**) macrophotographs taken with an optical microscope; (**c**,**d**) microphotographs taken with a scanning electron microscope.

**Figure 17:**
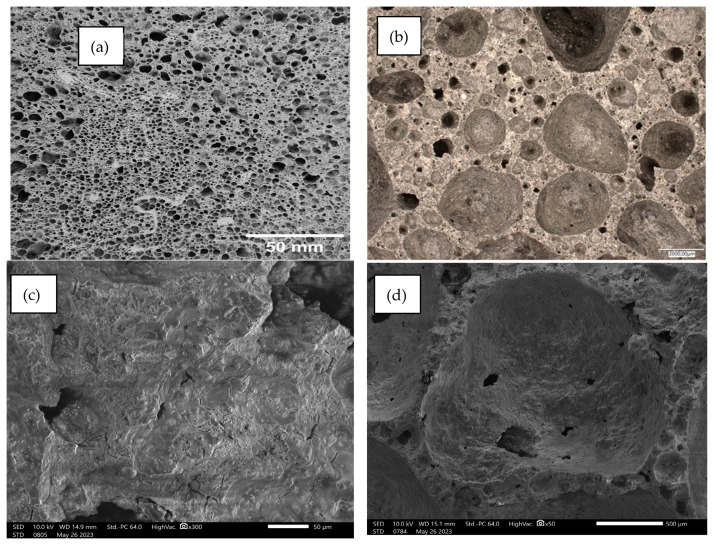
Images of the microstructure of foamed geopolymer 85FA_15BF: (**a**,**b**) macrophotographs taken with an optical microscope; (**c**,**d**) microphotographs taken with a scanning electron microscope.

**Figure 18:**
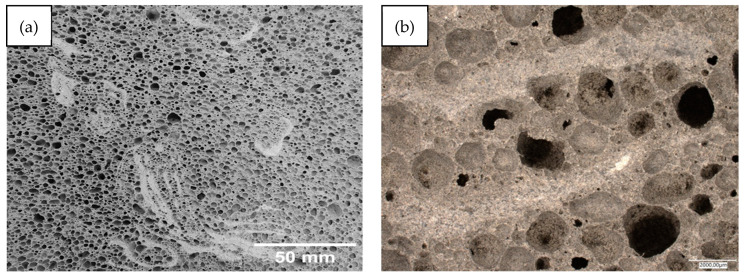

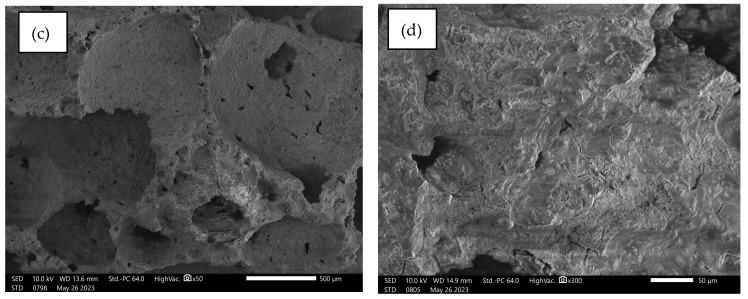
Images of the microstructure of foamed geopolymer 80FA_20BF: (**a**,**b**) macrophotographs taken with an optical microscope; (**c**,**d**) microphotographs taken with a scanning electron microscope.

Below, Figure 14, Figure 15, Figure 16, Figure 17 and Figure 18 present microstructural images taken with a scanning electron microscope and an optical microscope. All variants of the produced samples are shown in the figures. The images show a variety of pores of different shapes and sizes. In Figure 14, the largest pore size is characterized by the sample of the reference material (without basalt addition). The visualization of the porosity of the other samples is similar. The photographs also show different wall thicknesses forming the porous geopolymer structure. It can be unequivocally stated that the addition of basalt powder caused a decrease in the pore size of the geopolymer. As the amount of basalt powder addition increased, the number of smaller pores increased, and the number of large pores decreased. This is related to the change in the consistency of the geopolymer mixture due to the addition of basalt and the change in surface tension. This causes a greater number of small pores to be produced and large pores to be annihilated due to the release of the gases that constitute them.

**Figure 19:**
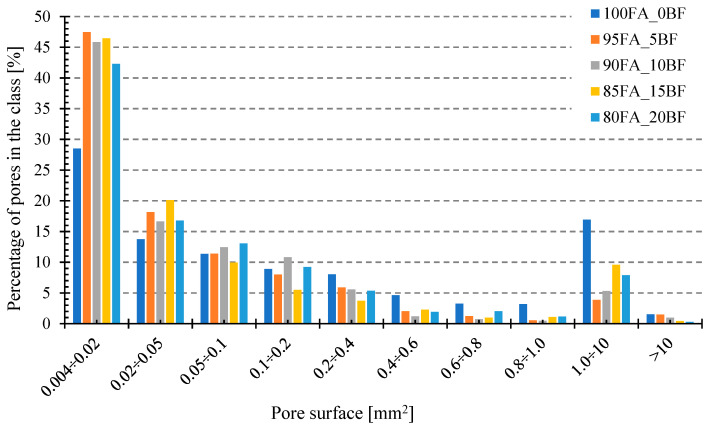
Dependence of the percentage of pores in classes on the pore area for geopolymer samples foamed with the addition of basalt.

Figure 19 below shows the results of the analysis of pore sizes in the tested samples. For the photos presented in Figure 14, Figure 15, Figure 16, Figure 17 and Figure 18a, analyses were carried out using the image analysis software “ImageJ” (https://imagej.net/ij/). The photos were properly cropped (to remove unnecessary background) and saved in shades of gray, which was necessary for analysis. Porosity was identified in the photos prepared in this way and, after appropriate graphic processing, the program calculated the average pore surface area. Samples made from mixtures with the addition of basalt flour were characterized by a much higher share of small pores and a less frequent occurrence of large pores, compared to the reference sample made from fly ash. The addition of basalt flour had a positive effect on reducing the average pore surface size compared to the reference sample 100FA_0BF.

## 4. Market Analysis and the Appropriateness of Using Basalt as an Addition to Geopolymers

The results presented in this paper illustrate the effect of basalt powder addition on the properties of foamed geopolymers. To date, basalt has been used in geopolymer materials in the form of reinforcing basalt fibers. However, it can also play the role of a precursor or stabilizing additive in the form of basalt powder with great success. Basalt processing plants report demand for basalt waste management in the form of powder or dust. This issue is particularly relevant nowadays, as many parts of the world are experiencing problems with the availability of coal combustion fly ash or smelter slag. The search for other waste sources of raw materials that can be precursors to geopolymers is of great importance.

Another very important problem that is currently causing difficulties in the implementation of geopolymer technology is the fact that most scientific studies are concerned with the use of fly ash and slag, which, as is well known, due to environmental policy and the shift away from coal-fired power generation, are running out, so their price is rising. It is now necessary to look anew for alternative waste sources other than by-products of coal combustion.

It is widely known that geopolymers can also be produced from available and chemically stable raw materials, such as metakaolins. However, it is important to note here the considerable cost of doing so. If implemented on a mass scale, products made from metakaolin-based geopolymers will not be price-competitive with traditional materials. The average cost of metakaolins in recent years has been about 400 USD per ton. 

Kaolin prices on world stock exchanges have fluctuated over the last 15 years at the level of USD 130 to USD 160 [44], while the prices of kaolin after calcination (metakaolin) range from USD 300 to USD 650 depending on the quality and degree of whiteness (for example, raw material prices available in China: [45]).

It turns out that in Poland (but also in other European countries), there is a systematic problem with the availability of ash. While the problem has been dealt with differently thus far, predictions for the future are not very optimistic. Even if fly ash becomes available, its price will rise—it is estimated that it will rise by as much as 1000%. Fly ash is an attractive material for the cement and concrete industries. There is already a shortage of ash, even for these industries. If there is a need to use them for geopolymers, the situation will become even worse.

**Figure 20:**
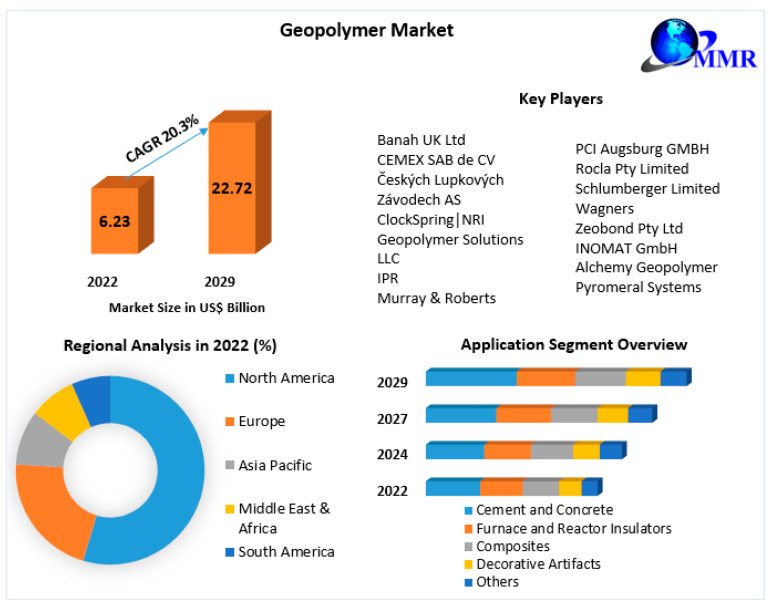
Geopolymer market forecasts to 2029 [46].

It is therefore necessary to look for other sources of raw materials for the production of geopolymers or to reduce the amount of fly ash used. The use of basalt is very attractive from this perspective. It is estimated that the geopolymer market, despite various difficulties, will develop very dynamically. Figure 20 presents forecasts for the geopolymer market (information provided at https://www.maximizemarketresearch.com/market-report/global-geopolymer-market/81356/ (accessed on 29 April 2024).

## 5. Conclusions

The studies conducted in connection with the introduction of an additive to foamed geopolymers in the form of basalt powder have led to the following conclusions:

1. As the amount of basalt powder added increases, the porosity structure changes. More spheroidal pores are formed and more pores of smaller sizes are created. This relationship was observed as a result of microscopic observations. This is probably related to the change in the consistency of the geopolymer mixture and its surface tension, which results in the formation of pores of different sizes more than in the case of a geopolymer mixture without the addition of basalt.
2. The addition of basalt powder has a positive effect on the stability of foamed structures and the ability to foam the geopolymer mass. The addition of 5% basalt powder causes an increase in density by 14%, as well as an increase in compressive strength by 63% and an increase in bending strength by 20%. The insulating properties due to the addition of 5% basalt powder decreased only by less than 7%. These dependencies result from the change in the consistency of the geopolymer mixture with the addition of basalt. The addition of 5% basalt is the optimal proportion of this addition because the highest increase in compressive strength was achieved with a slight deterioration of insulating properties (by 7%) despite an increase in density by 14%.
3. Further research should be carried out to optimize the share of basalt powder and optimize the molar ratios of activating solutions, taking into account that some of the basalt is reactive and takes part in the geopolymerization reaction and does not only act as a filler. The addition of basalt powder to geopolymers can contribute to the improvement of several parameters, such as: improvement of strength properties and abrasion resistance; improvement of thermal and insulating properties; increase in the ability to accumulate energy; porosity reduction; increased corrosion resistance in aggressive environments; reducing shrinkage and the risk of cracking. The ecological aspect is also important here because it is possible to use basalt in crushed form as waste. The effectiveness of the addition of powdered basalt will depend on many factors, including the origin of this material and its chemical and phase composition. Undoubtedly, further work should be carried out and the proportions of basalt addition should be adjusted to specific application requirements. It is also inevitable to take into account and accept a certain compromise because it is known that the addition of basalt will not improve all properties.

## Figures and Tables

**Table 1:** XRF oxide analysis of fly ash (main ingredients only).

Precursor	Oxide Composition (wt.%)						
	SiO_2_	TiO_2_	Fe_2_O_3_	Al_2_O_3_	CaO	MgO	K_2_O
Fly ash	56.64 ± 0.32	0.87 ± 0.01	4.99 ± 0.01	30.57 ± 0.41	2.79 ± 0.02	0.1 ± 0.01	2.74 ± 0.01
Basalt	46.92 ± 0.10	2.66 ± 0.03	17.48 ± 0.01	13.01 ± 0.08	10.55 ± 0.01	5.95 ± 0.11	1.20 ± 0.01

**Table 2:** Parameters of grain size of raw materials used for testing.

		D₁₀ [μm]	D₅₀ [μm]	D₉₀ [μm]	Average Value [μm]
Fly ash	Average value	2.09	11.00	25.42	13.26
	Standard deviation	0.08	0.21	0.76	0.30
Basalt powder	Average value	2.62	20.48	55.71	26.72
	Standard deviation	0.01	0.14	0.30	0.15

**Table 3:** Variants of geopolymer compositions prepared for testing.

Sample ID	Mass Share [%]	
Fly Ash	Basalt Powder	
100FA_0BF	100	0
95FA_5BF	95	5
90FA_10BF	90	10
85FA_15BF	85	15
80FA_20BF	80	20

**Table 4:** Additional components of geopolymer mixtures used in each composition variant.

Components of the Geopolymer Mix	Amount per 1000 g of Fly Ash
Gypsum	100 g
Cellulose	5 g
Alkaline solution	440 mL
Hydrogen peroxide	30 mL

**Table 5:** Flexural and compressive strength values for foamed geopolymers with basalt powder.

	100FA_0BF	95FA_5BF	90FA_10BF	85FA_15BF	80FA_20BF
Compressive strength [MPa]	0.89	1.45	0.91	0.84	0.71
Flexural strength [MPa]	0.56	0.67	0.45	0.40	0.34

## Data Availability

Data are contained within the article.
